# Percutaneous ethanol injection for hepatocellular carcinoma: 20-year outcome and prognostic factors

**DOI:** 10.1111/j.1478-3231.2012.02838.x

**Published:** 2012-06-19

**Authors:** Shuichiro Shiina, Ryosuke Tateishi, Masatoshi Imamura, Takuma Teratani, Yukihiro Koike, Shinpei Sato, Shuntaro Obi, Fumihiko Kanai, Naoya Kato, Haruhiko Yoshida, Masao Omata, Kazuhiko Koike

**Affiliations:** Department of Gastroenterology, University of TokyoTokyo, Japan

**Keywords:** ablation, hepatocellular carcinoma, percutaneous ethanol injection, prognostic factor, recurrence, survival, treatment outcome

## Abstract

**Background:**

Ethanol injection is the best-known image-guided percutaneous ablation for hepatocellular carcinoma (HCC) and a well-tolerated, inexpensive procedure with few adverse effects. However, there have been few reports on its long-term results.

**Aims:**

We report a 20-year consecutive case series at a tertiary referral centre.

**Methods:**

We performed 2147 ethanol injection treatments on 685 primary HCC patients and analysed a collected database.

**Results:**

Final computed tomography demonstrated complete ablation of treated tumours in 2108 (98.2%) of the 2147 treatments. With a median follow-up of 51.6 months, 5-, 10- and 20-year survival rates were 49.0% [95% confidence interval (CI) = 45.3–53.0%], 17.9% (95% CI = 15.0–21.2%) and 7.2% (95% CI = 4..5–11.5%) respectively. Multivariate analysis demonstrated that age, Child–Pugh class, tumour size, tumour number and serum alpha-fetoprotein level were significant prognostic factors for survival. Five-, 10- and 20-year local tumour progression rates were 18.2% (95% CI = 15.0–21.4%), 18.4% (95% CI = 15.2–21.6%) and 18.4% (95% CI = 15.2–21.6%) respectively. Five-, 10- and 20-year distant recurrence rates were 53.5% (95% CI = 49.4–57.7%), 60.4 (95% CI = 56.3–64.5%) and 60.8% (95% CI = 56.7–64.9%) respectively. There were 45 complications (2.1%) and two deaths (0.09%).

**Conclusions:**

Ethanol injection was potentially curative for HCC, resulting in survival for more than 20 years. This study suggests that new ablation therapies will achieve similar or even better long-term results in HCC.

Hepatocellular carcinoma (HCC) is the fifth most common malignant neoplasm in the world. Only 20% of HCC patients are candidates for resection 1. Furthermore, recurrence is frequent even after curative resection. Liver transplantation is restricted by donor shortage. Thus, various non-surgical therapies have been introduced 2. Among these, image-guided percutaneous ablation is considered best for early-stage HCC.

The most studied percutaneous ablation is ethanol injection. Ethanol injection is a well-tolerated, inexpensive procedure with few adverse effects and has been considered the standard against which any new ablation therapy should be compared 2. Although ethanol injection was introduced into clinical practice in the 1980s #b^3^b^4^, few reports of its long-term results have been published #b^5^b^6^b^7^b^8^. We report here a 20-year consecutive case series at a tertiary referral centre. This study documents the largest number of ethanol injection treatments at a single institution. Findings in this 20-year experience may be extrapolated to other ablation therapies, such as radiofrequency ablation, in which such long-term outcomes are not yet available 9.

## Patients and methods

### Indications for ethanol injection

Ethanol injection was performed in patients satisfying the following criteria: (i) ineligible for resection or transplantation, or had refused surgery; (ii) no extrahepatic metastasis or vascular invasion. Exclusion criteria were as follows: (i) tumour was not visualized by ultrasonography or not accessible percutaneously; (ii) total bilirubin level ≥3.0 mg/dl; (iii) platelet count <40 × 10^9^/L; (iv) prothrombin activity <35%; (v) refractory ascites. In general, we performed ethanol injection on patients with Child–Pugh class A or B, with 3 or fewer tumours ≤3 cm in diameter. We performed ethanol injection on patients beyond these conditions, however, who were likely to benefit from the procedure for possible cure or prolongation of life. No patients were excluded solely because of tumour location 10. Informed consent was obtained from each patient. This study was conducted according with the Helsinki Declaration of 1975 and approved by the Institutional Review Board.

### Patients

**Fig. 1:**
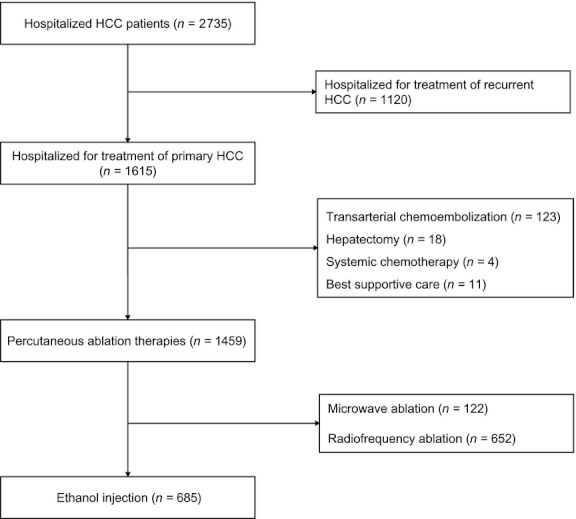
Flow of patients in this study. HCC, hepatocellular carcinoma

In this cohort study, we analysed a prospectively collected computerized database. Between 1985 and 2005, 2735 HCC patients were admitted to the Department of Gastroenterology, University of Tokyo (Fig. 1). At initial hospitalization, 1615 had primary HCC and the remaining 1120 had recurrent HCC. The recurrent HCC patients had undergone therapies other than ethanol injection for primary HCC.

Of the 1615 patients with primary HCC, 1459 (90.3%) underwent percutaneous ablation as the initial treatment, including ethanol injection. The remaining 156 patients received other therapies: transarterial chemoembolization for 123 patients with multinodular or large tumours that could not be treated by ablation therapies; hepatic resection for 18 with good liver function who consented to an operation; chemotherapy for four with vascular invasion or extrahepatic metastasis; and best supportive care for 11 with decompensated cirrhosis or poor general condition.

Of the 1459 patients treated by percutaneous ablation, 685 underwent ethanol injection, 122 underwent microwave ablation, and the remaining 652 radiofrequency ablation. The type of percutaneous ablation performed varied with the date of treatment. We started ethanol injection in December 1985, microwave ablation in October 1995 and radiofrequency ablation in February 1999 11. Between October 1995 and February 1999, both ethanol injection and microwave ablation were performed. Microwave ablation was chosen for patients who had better liver function and whose tumour was located in a position where the electrode could be inserted and held safely. Since February 1999, both ethanol injection and radiofrequency ablation have been performed. Between April 1999 and January 2001, 232 patients with three or fewer tumours, each ≤3 cm in diameter, and Child–Pugh class A or B were entered into a randomized controlled trial 12. Patients outside these inclusion criteria were mostly treated by radiofrequency ablation. After this trial, radiofrequency ablation was generally the treatment of choice, and ethanol injection was used only in those unsuitable for radiofrequency ablation: those with either enterobiliary reflux or tumour adhesion to the gastrointestinal tract.

Hepatocellular carcinoma was diagnosed based on typical imaging findings of early phase enhancement and late phase contrast washout on computed tomography (CT) 13. HCC diagnosis was also confirmed by biopsy in 630 (92.0%) of the 685 patients with primary HCC treated by ethanol injection. A total of 587 (85.7%) were diagnosed as having cirrhosis.

In general, chemoembolization was combined with ethanol injection in patients with either ≥4 tumours or those with two or three tumours at least one of which is >3.0 cm in diameter. The combination of chemoembolization with ethanol injection was performed in 186 patients.

### Treatment methods

Preoperative planning including ultrasound examination and evaluation of all imaging findings was performed to identify the tumours and to determine the access route. The procedure was performed according to an institutional protocol and under the supervision of experienced physicians who had performed this treatment more than 200 times. The precise techniques of ethanol injection are described elsewhere 12. Briefly, all procedures were performed percutaneously under ultrasound guidance. Artificial pleural effusion or artificial ascites method is much less frequently used in ethanol injection compared with radiofrequency ablation, because the procedure is necessary to be repeated several times. Since 1990, we have used two or three needles to inject ethanol into several sites in one procedure 12. Ethanol injection was performed twice per week. The procedure was repeated until ethanol appeared to have been injected throughout the tumour. To judge a timing to stop repetition of injecting ethanol and to order a CT scan, we considered total volume of injected ethanol and change of echogenicity. The general guideline for the necessary volume of injected ethanol was calculated according to the following numerical expression, V = (4/3) π (r + 0.5)^3^, where V (in millilitres) is the volume of ethanol and r (in centimetres) is the radius of the tumour; 0.5 is added to provide a safety margin, which is based on the concept that some surrounding liver parenchyma all around the tumour as well as the tumour itself must be ablated 5.

A CT scan was then performed 1–3 days after the procedure to evaluate technique effectiveness 14. Complete ablation was defined as hypoattenuation of the entire tumour. When the presence of unablated tumour portions was suspected, a few more procedures were performed. We did not predefine the number of procedures in a treatment. The ethanol injection treatment was generally continued until CT demonstrated the entire tumour necrosis.

### Follow-up

Follow-up investigations consisted of CT, ultrasonography and measurement of serum α-fetoprotein (AFP), des-γ-carboxy-prothrombin (DCP) (since April 1993) levels and lectin-reactive AFP (AFP-L3) (since July 1997) every 4 months. Local tumour progression was defined as appearance of viable tumour touching the original tumour 14 and distant recurrence as emergence of tumour(s) separate from the primary site. Ethanol injection was used for recurrence if the patient still met the indication criteria. If multiple recurrences were not treatable with ethanol injection, chemoembolization was generally performed.

### Statistical analyses

This study is a report of a consecutive case series. All ethanol injection treatments performed on primary HCC patients at the Department of Gastroenterology, University of Tokyo between 1985 and 2005 were included. Data are presented as mean ± SD for quantitative variables, and as absolute frequencies for qualitative variables.

A ‘procedure’ was defined as a single intervention episode that consisted of one or more ablations performed on tumours, and a ‘treatment’ as the completed effort to ablate tumours. A treatment consisted of several procedures 14. ‘Technique effectiveness’ rate was defined as the percentage of successfully eradicated macroscopic tumours as evidenced at CT scan after the last procedure 14. In cases in which there was Lipiodol deposit inside the tumour because of the combination of chemoembolization with ethanol injection, we judged that the tumour had been successfully eradicated if it was surrounded with completely non-enhanced tissue in final CT.

Overall survival was calculated in the 685 primary HCC patients. Survival curves were generated using the Kaplan–Meier method. In addition to overall survival, subgroup analyses were performed with clinical characteristics including tumour size, tumour number and Child–Pugh class. Recurrence was evaluated in 591 patients in whom ethanol injection was performed with curative intent. All tumours were treated by ethanol injection in those patients. The remaining 94 patients were excluded from the recurrence analysis because some small tumours had been left untreated by ethanol injection on account of detection failure by ultrasonography. Recurrence rates were calculated using the Gaynor method 15. All time estimates were made from the date of the first ethanol injection. The follow-up was finalized at either death or the last visit to the outpatient clinic before December 31 2010. Transplanted patients were censored from this study at the date of transplantation.

The prognostic relevance of baseline variables (Table 1), the combination of chemoembolization, HCC recurrence and the number of ethanol injection sessions to survival was analysed by univariate and multivariate models. The prognostic relevance of baseline variables (Table 1), the combination of chemoembolization and the number of ethanol injection sessions to local tumour progression and distant recurrence was also analysed by univariate and multivariate models. In multivariate analysis, we evaluated models including Child–Pugh class and excluding its components to avoid multicollinearity. Serum DCP and AFP-L3 levels were excluded from the multivariate model because of absence of data from 168 and 461 patients respectively. Some continuous variables in which log-linearity could not be assumed were transformed into categorical variables. Variables with a *P* value <0.05 determined by univariate comparison were subjected to multivariate analysis. A stepwise variable selection was performed with Akaike Information Criteria in multivariate analysis. Results were expressed as hazard ratios with corresponding 95% confidence intervals (CI), with *P* values from the Wald test. All significance tests were two-tailed, and differences with a *P* value <0.05 were considered statistically significant.

**Table 1:** Baseline characteristics of the 685 Patients undergoing percutaneous ethanol injection for primary hepatocellular carcinoma

Variable	
Age (years)	64.0 ± 8.9
Males, *n* (%)	502 (73.3)
Viral infection*	
HBs-Ag positive, *n*/*N* (%)	64/685 (9.3)
Anti-HCV positive, *n*/*N* (%)	570/673 (84.7)
Both positive, *n*/*N* (%)	11/673 (1.6)
Both negative, *n*/*N* (%)	52/673 (7.7)
Alcohol consumption >80 g/day, *n* (%)	143 (20.9)
Ascites, *n* (%)	122 (17.9)
Encephalopathy, *n* (%)	44 (6.5)
Albumin (g/dl)	3.55 ± 0.50
Total bilirubin (mg/dl)	0.96 ± 0.536
Prothrombin time (%)	71.6 ± 15.9
Platelet count (×10^4^/mm^3^)	10.3 ± 4.6
AST (IU/L)	80.6 ± 48.2
ALT (IU/L)	79.2 ± 61.9
Child–Pugh class, *n* (%)	
A	425 (62.1)
B	228 (33.3)
C	32 (4.6)
Tumour size (cm)	2.83 ± 1.47
Tumour number	2.0 ± 1.7
Serum AFP (ng/ml), *n* (%)	
≤100	525 (76.6)
101–400	95 (13.9)
>400	65 (9.5)
Serum DCP (mA U/ml), *n* (%)†	
≤100	428 (82.8)
101–400	49 (9.5)
>400	40 (7.7)
Serum AFP-L3 (%), *n* (%)‡	
≤15	193 (86.2)
15.1–40	16 (7.1)
>40	15 (6.7)

* Anti-HCV was not tested in 12 patients.

† Serum DCP level was not measured in 168 patients.

‡ Serum AFP-L3 level was not measured in 461 patients.

HBs-Ag, hepatitis B surface antigen; HCV, hepatitis C virus; AFP, α-fetoprotein; DCP, des-gamma-carboxy-prothrombin; AFP-L3, lectin-reactive α-fetoprotein.

Data are expressed as mean ± standard deviation.

Complications were defined according to the guidelines of the Society of Interventional Radiology 16.

## Results

### Antitumour effect

We performed 2147 ethanol injection treatments, comprising 13 526 procedures. Thus, procedure number per treatment was 6.3 ± 2.6. The total volume of injected ethanol per treatment was 40.9 ± 16.3 ml. Many patients received iterative ethanol injection treatments for recurrence. A total of 108 patients underwent ethanol injection treatment once, 118 patients twice, 196 patients 3 times, 153 patients 4 times, 71 patients 5 times, 28 patients 6 times, 8 patients 7 times and 3 patients 8 times.

Technique effectiveness rate was 98.2% (2108/2147 treatments). It was similar between the initial ethanol injection treatments and the other ethanol injection treatments for recurrence (*P* = 0.397). Complete ablation of the tumour was achieved in 675 (98.5%) of the 685 initial treatments and in 1433 (98.0%) of the 1462 other treatments. However, technique effectiveness rate significantly differed with tumour size (*P* = 0.002). No apparent viable portions remained in 758 (99.0%) of 766 treatments for tumours ≤2.0 cm in diameter, in 704 (98.4%) of 717 treatments for tumours 2.1–3.0 cm, in 570 (97.9%) of 582 treatments for tumours 3.1–5.0 cm and in 76 (92.7%) of 82 treatments for tumours >5.0 cm.

### Survival

Table 1 shows clinical characteristics of the 685 patients. A total of 136 patients (19.9%) were older than 75 years. In all, 180 patients had tumours ≤2.0 cm in diameter, 274 had tumours 2.1–3.0 cm, 192 had tumours 3.1–5.0 cm and 39 had tumours >5.0 cm. A total of 367 patients had one tumour, 238 patients had 2 or 3 tumours and 80 had 4 or more tumours.

As of December 2010 (with a median follow-up of 51.6 months), 70 patients (10.2%) remained alive, 52 (7.6%) were lost to follow-up and 563 (82.2%) had died. Of the 685 patients, two were transplanted. The number of patients who survived longer than 5, 10 and 20 years after the first ethanol injection treatment was 305, 97 and 3 respectively. The cause of death was HCC in 297 patients (52.8%), liver failure in 129 (22.9%), upper gastrointestinal bleeding in 30 (5.3%), complications related to the procedure in 2 (0.4%), liver-unrelated diseases in 84 (14.9%) and undetermined in 21 (3.7%).

**Fig. 2:**
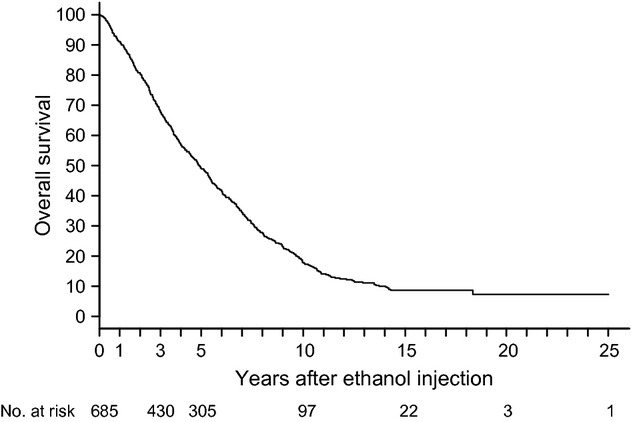
Overall survival in 685 primary hepatocellular carcinoma patients who underwent ethanol injection.

The 1-, 3-, 5-, 10-, 15- and 20-year survival rates of all 685 patients were 91.0% (95% CI = 88.9–93.2%), 67.6% (95% CI = 64.1–71.3%), 49.0% (95% CI = 45.3–53.0%), 17.9% (95% CI = 15.0–21.2%), 8.6% (95% CI = 6.4–11.7%) and 7.2% (95% CI = 4.5–11.5%) respectively (Fig. 2; Table 2). Survival rates significantly differed with tumour number (*P* = 0.0001), tumour size (*P* = 0.0001) and Child–Pugh class (*P* = 0.0001). In patients with 1–3 tumours, all ≤3 cm, and in Child–Pugh class A or B, the 5-year survival rate was 59.5% (95% CI: 54.7–64.7%).

**Table 2:** Survival of patients undergoing ethanol injection, based on tumour number, tumour size and Child–Pugh class

		Survival (%)						
				
Grading	*n*	3-Year	5-Year	10-Year	15-Year	20-Yar	Median (years)	*P* value
Overall survival	685	67.6	49.0	17.9	8.6	7.2	4.9	–
Tumour number								
Solitary	367	72.0	56.5	24.6	12.1	9.7	5.8	0.0001
2–3	232	71.5	46.3	12.9	5.9	–	4.7	
≥4	86	37.6	23.8	2.5	1.3	–	2.6	
Tumour size								
≤2.0 cm	240	83.6	63.8	27.6	12.3	6.1	6.9	0.0001
2.1–3.0 cm	221	68.0	47.9	15.0	10.7	10.7	4.8	
>3.0 cm	224	50.2	34.4	10.1	3.5	3.5	3.1	
Child–Pugh class								
A	425	77.3	58.7	24.4	12.5	10.4	6.2	0.0001
B	228	53.9	35.5	8.1	3.0	–	3.5	
C	32	37.5	18.8	3.1	–	–	1.9	
Combination of tumour number, tumour size, and Child–Pugh class								
Solitary, ≤3 cm	275	77.5	62.2	28.8	14.5	10.8	6.8	–
Solitary, ≤3 cm, Child–Pugh A	185	84.9	69.2	36.7	20.2	15.1	7.6	–
1–3 tumours, ≤3 cm	419	78.6	58.0	23.5	12.2	9.1	6.1	–
1–3 tumours, ≤3 cm, Child–Pugh A/B	402	80.5	59.5	24.3	12.8	9.6	6.2	–
Satisfied the indication criteria of surgical resection proposed in the BCLC protocol*	121	86.3	72.8	31.1	14.8	–	7.2	–

* Child–Pugh class A with a normal level of bilirubin, no significant portal hypertension and a single HCC.

BCLC, Barcelona Clinic Liver Cancer; HCC, hepatocellular carcinoma.

Univariate analysis indicated that 13 of the 22 variables were relevant to survival. In multivariate analysis, a model that contained age, antibody to hepatitis C virus (anti-HCV), Child–Pugh class, tumour size, tumour number and serum AFP level was selected (Table 3).

**Table 3:** Multivariate analysis of variables relevant to survival, local tumour progression and distant recurrence

Variable	Multivariate analysis Hazard ratio (95% CI)	*P* value
Survival		
Age (per year)	1.03 (1.02–1.04)	<0.0001
Anti-HCV-positive	0.81 (0.69–0.94)	0.006
Child–Pugh class		
A	1	
B	2.01 (1.66–2.44)	<0.0001
C	3.11 (2.08–4.65)	<0.0001
Tumour size (cm)		
≤2.0	1	
2.1–3.0	1.26 (1.00–1.58)	0.051
3.1–5.0	1.51 (1.18–1.93)	0.001
>5.0	2.31 (1.61–3.31)	<0.0001
Tumour number		
solitary	1	
2–3	1.10 (0.90–1.35)	0.34
≥4	2.11 (1.59–2.78)	<0.0001
Serum AFP (ng/dl)		
≤100	1	
101–400	1.47 (1.14–1.90)	0.003
>400	2.16 (1.57–2.97)	<0.0001
Local tumour progression		
Tumour size (cm)		
≤2.0	1	
2.1–3.0	1.47 (1.15–1.88)	0.002
3.1–5.0 vs. ≤2.0	1.30 (0.97–1.75)	0.08
>5.0 vs. ≤2.0	2.81 (1.64-4.82)	0.0002
Distant recurrence		
Tumour size (cm)		
≤2.0	1	
2.1–3.0	1.42 (1.11–1.82)	0.006
3.1–5.0	1.28 (0.95–1.72)	0.10
>5.0	2.48 (1.43–4.28)	0.001
Tumour number		
solitary	1	
2–3	1.47 (1.16–1.85)	0.001
≥4	2.12 (1.36–3.28)	0.0008

AFP, α-fetoprotein; CI, confidence interval; HCV, hepatitis C virus.

**Fig. 3:**
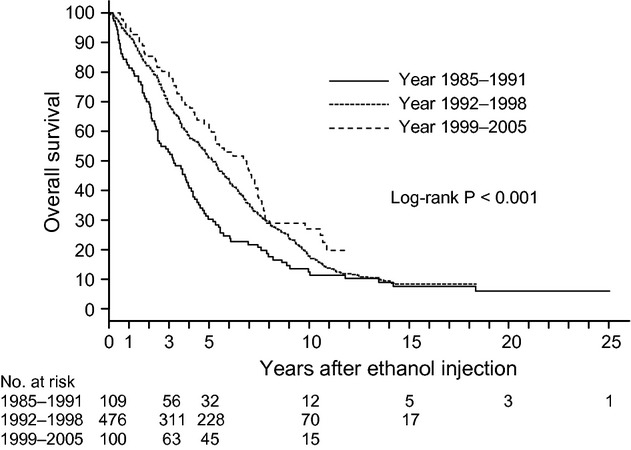
Survival according to the time period in which the first ethanol injection was performed (1985–1991 vs. 1992–1998 vs. 1999–2005)

Survival rates significantly differed with the time period in which the first ethanol injection was performed (*P* < 0.0001; Fig. 3). In 109 patients who underwent ethanol injection between 1985 and 1991, the 5-year survival rate was 30.3% (95% CI = 22.7–40.5%), whereas it was 51.2% (95% CI = 46.8–55.9%) in 476 patients between 1992 and 1998, and 61.1% (95% CI = 51.3–72.8%) in 100 patients between 1999 and 2005.

### Recurrence

Recurrence developed in 449 patients. Local tumour progression alone was found in 61 patients, local tumour progression with distant recurrence in 44 and distant recurrence alone in 344. Of these 344 patients, eight had recurrence in extrahepatic sites: five had lymph node metastasis, one had lung metastasis, one had bone metastasis and the remainder had both lymph node and lung metastasis. Of the 449 patients, the first recurrence was treated by iterative ethanol injection in 399 (88.8%), chemoembolization in 44 (9.8%), systemic chemotherapy in three (0.7%) and best supportive care in three (0.7%).

**Fig. 4:**
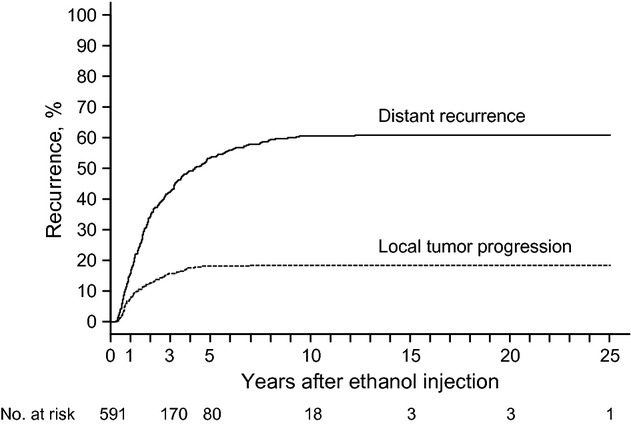
Local tumour progression or distant recurrence in patients who underwent ethanol injection.

The 1-, 3-, 5-, 10-, 15- and 20-year rates of local tumour progression with or without distant recurrence were 7.9% (95% CI = 5.7–10.0%), 15.6% (95% CI = 12.6–18.6%), 18.2% (95% CI = 15.0–21.4%), 18.4% (95% CI = 15.2–21.6%), 18.4% (95% CI = 15.2–21.6%) and 18.4% (95% CI = 15.2–21.6%) respectively (Fig. 4). Univariate analysis demonstrated that three variables were relevant to local tumour progression, whereas multivariate analysis indicated that only tumour size was significantly related to local tumour progression (Table 3).

The 1-, 3-, 5-, 10-, 15- and 20-year rates of distant recurrence without local tumour progression were 17.1% (95% CI = 14.0–20.1%), 42.6% (95% CI = 38.6–46.7%), 53.5% (95% CI = 49.4–57.7%), 60.4% (95% CI = 56.3–64.5%), 60.8% (95% CI = 56.7–64.9%) and 60.8% (95% CI = 56.7–64.9%) respectively. Univariate analysis demonstrated that five variables were relevant to distant recurrence, whereas multivariate analysis indicated that tumour size and tumour number were significantly related to distant recurrence without local recurrence (Table 3).

### Complications

Table 4 shows complications encountered. The incidence rates per treatment and per procedure were 2.1% (45 of 2147) and 0.33% (45 of 13 526) respectively. A patient died of multiple organ dysfunction syndrome caused by procedure-related hemoperitoneum. The tumour was not on the surface but inside the liver. The patient did not have marked bleeding tendency. The other developed myocardial infarction, resulting in death during the procedure. The treatment mortality rate was 0.06%.

**Table 4:** Complications in 2147 treatments of ethanol injection for hepatocellular carcinoma

Complication	Number
Neoplastic seeding	9
Hemoperitoneum	9
Hemobilia	6
Liver abscess	6
Symptomatic pleural effusion	3
Massive hepatic infarction	3
Biliary cast	2
Hemothorax	2
Abnormal decrease in blood coagulation factor VIII	2
Biloma	1
Biliary bronchial fistula	1
Myocardial infarction	1

## Discussion

This study describes a 20-year experience with ethanol injection at a high-volume centre. We performed 2147 ethanol injection treatments on the 685 primary HCC patients, showing that ethanol injection has a high antitumour effect. Tumours were judged to have been completely ablated by final CT imaging in 98.2% of the treatments. The complete response rate may be higher in this study than others #b^17^b^18^, probably because we did not predefine the number of procedures in a treatment. We generally repeated the procedure until CT demonstrated complete tumour necrosis. Many other studies limited the procedure number of ethanol injection. Complete tumour ablation has been reported to relate to improved survival 19.

This study showed that ethanol injection could achieve long-term survival over 20 years. Ninety-seven patients survived for more than 10 years and three for more than 20 years. Both tumour factors and liver function were relevant to survival. In addition, age was among the prognostic factors. In this study, 19.9% were older than75 years, which may have resulted in the higher percentage (14.9%) of liver-unrelated deaths compared with other studies. Ant-HCV positivity was a good prognostic factor in this study.

Survival in ethanol injection appears to have improved with times. This is probably because of advances in imaging techniques, such as ultrasound and CT, more refined skills and greater experience in ablation and innovations in the treatment of underlying liver diseases.

Hepatocellular carcinoma frequently recurred after ethanol injection. Most recurrences were, however, not local tumour progression but distant recurrence. Frequent recurrence is not specific to ethanol injection. After hepatic resection, the tumour recurrence rate exceeds 70% at 5 years #b^20^b^21^. In this study, periodic follow-up detected most recurrence at limited stage. Ethanol injection was performed again for first recurrence in 88.8% of the cases. In hepatic resection, the rate of repeat resection for first recurrence has been reported to range from 10.4 to 30.6% #b^21^b^22^. As ethanol injection is less invasive than hepatic resection, iterative ethanol injection can be performed for recurrence more easily.

Ethanol injection was a safe procedure, although many patients in this study were at risk for surgical treatment because of advanced cirrhosis or other comorbidities. Only 121 (17.7%) of the 685 patients satisfied the indication criteria of surgical resection proposed in the BCLC (Barcelona Clinic Liver Cancer) protocol 23 and were, thus, considered good candidates for surgical resection. Other investigators also reported low complication rates of 0–3.2% #b^6^b^7^b^8^b^24^. For hepatic resection, morbidity rates have been reported to be 38–47% even in recent studies #b^25^b^26^b^27^.

Radiofrequency ablation has steadily replaced ethanol injection 11. At our institution, radiofrequency ablation is currently the first option for percutaneous ablation 28. Several randomized controlled trials including ours #b^12^b^18^b^29^b^30^ demonstrated more reliable local antitumour effect and higher survival. Our 10-year outcome of radiofrequency ablation 28 appears superior to this 20-year outcome of ethanol injection. In addition, radiofrequency ablation requires fewer treatment sessions and shorter hospitalization.

A meta-analysis showed, however, that ethanol injection did not differ from radiofrequency ablation for tumours ≤2 cm in diameter 31. A recent randomized controlled trial also demonstrated similar 5-year survival between the two ablations 32. Ethanol injection is at least more feasible and cheaper than radiofrequency ablation.

Surgical resection has been considered the treatment of first choice for HCC. Our first option for resectable tumours was also surgery. However, most patients who came to our department declined surgical resection. Thus, some patients in this study underwent ethanol injection not because of unresectable tumour but because of refusal of surgery. Those who preferred surgery would have gone directly to the surgical department, which has extensive experience in hepatic resection 27.

It is not easy to compare outcomes between ethanol injection and surgical resection. Indications are different between the two treatments. Furthermore, indications for each treatment are different from institution to institution. Thus, a case adjudged to be treatable by ethanol injection or surgical resection at an institution may not be given the same treatment at another. The best-known indication criteria may be those proposed in the BCLC protocol 23, which states that surgical resection should be restricted to patients with performance status 0, Child–Pugh class A, single HCC, normal portal pressure and normal serum bilirubin level. In patients satisfying those criteria, the 5-year survival rate is expected to be >70% 20. In this study, 5-year survival rate of the patients satisfied the criteria was 72.8%, which appears satisfactory when compared with outcomes following surgical resection. Furthermore, in patients with solitary HCC, ≤3 cm in diameter, and Child–Pugh A, 5- and 10-year survival rates were 69.2% and 36.7% respectively. In patients treated by surgical resection, 5- and 10-year survival rates were 34.4–70.0% and 10.5–52.0% respectively #b^22^b^33^b^34^b^35^b^36^b^37^b^38^b^39^. Although this is an observational study with no control, survivals following ethanol injection appear comparable to those reported following surgical resection.

A randomized controlled trial showed no significant difference in survival between ethanol injection and surgical resection #b^40^. Several non-randomized controlled trials also reported similar overall survival between the two treatments #b^5^b^6^b^7^b^40^b^41^b^42^b^43^, whereas others reported higher survival with resection #b^44^. Further studies are necessary to resolve this issue of comparing ablation with resection.

We made strenuous efforts to standardize the procedure of ethanol injection because many physicians performed ethanol injection at our institution. In addition to proficient practice of ethanol injection, detailed preoperative planning, cautious postoperative evaluation of therapeutic effect and careful follow-up are vital to achieve satisfactory outcomes.

Source population in this study may represent selection bias, as we performed ethanol injection on most patients who were hospitalized at our department; however, many patients with unfavourable tumour conditions for ethanol injection might not have been referred to us. Therefore, caution is required when extrapolating our findings to the general population of HCC patients.

A second limitation is that study population cannot be clearly defined. This study was based on daily clinical practice over a 20-year period. Indication criteria of ethanol injection changed over time, mainly because of the introduction of the other ablations: microwave ablation and radiofrequency ablation. Furthermore, various treatments besides percutaneous ablations were available for HCC, such as surgical resection and chemoembolization, with frequently overlapping indications.

One further limitation is the fact that this was a single-centre study. To extrapolate the findings in this study to patients at other institutions, consideration should be given to differences in the indications, methods, expertise, performance of available ultrasound and CT equipment and others. Treatment outcome may be influenced by the physicians’ expertise and the institution's volume of care. We performed over 2000 ethanol injection treatments, which may represent a much greater number of treatments than those in most other institutions.

In conclusion, our 20-year experience shows that ethanol injection was potentially curative, resulting in long-term survival over 20 years. Findings in this study may suggest that other ablation therapies, such as radiofrequency ablation, will achieve similar or even better long-term results in HCC.

## Abbreviations

AFP-L3: lectin-reactive AFP
AFP: α-fetoprotein
BCLC: Barcelona Clinic Liver Cancer
CI: confidence intervals
CT: computed tomography
DCP: des-γ-carboxy-prothrombin
HBs-Ag: hepatitis B surface antigen
HCC: hepatocellular carcinoma
HCV: hepatitis C virus
